# Increasing the Efficacy of Oncolytic Adenovirus Vectors

**DOI:** 10.3390/v2091844

**Published:** 2010-08-27

**Authors:** Karoly Toth, William S. M. Wold

**Affiliations:** Department of Molecular Microbiology and Immunology, Saint Louis University School of Medicine, 1100 S. Grand Blvd, St. Louis, MO 63104, USA; E-Mail: woldws@slu.edu

**Keywords:** adenovirus, replication competent, tumor, virotherapy

## Abstract

Oncolytic adenovirus (Ad) vectors present a new modality to treat cancer. These vectors attack tumors via replicating in and killing cancer cells. Upon completion of the vector replication cycle, the infected tumor cell lyses and releases progeny virions that are capable of infecting neighboring tumor cells. Repeated cycles of vector replication and cell lysis can destroy the tumor. Numerous Ad vectors have been generated and tested, some of them reaching human clinical trials. In 2005, the first oncolytic Ad was approved for the treatment of head-and-neck cancer by the Chinese FDA. Oncolytic Ads have been proven to be safe, with no serious adverse effects reported even when high doses of the vector were injected intravenously. The vectors demonstrated modest anti-tumor effect when applied as a single agent; their efficacy improved when they were combined with another modality. The efficacy of oncolytic Ads can be improved using various approaches, including vector design, delivery techniques, and ancillary treatment, which will be discussed in this review.

## 1. Introduction

In spite of tremendous advances in the prevention, diagnosis, and therapy of cancer, it is the second most frequent cause of mortality in the United States. Many forms of cancers are considered to be incurable, and treatment for many tumors results in only a minor increase in the time of survival. Further, these treatment regimes cause serious side effects; some patients decide to receive palliative treatment only. It is not surprising that researchers continuously work to develop new modalities for anti-cancer therapy.

One such new method uses genetically modified oncolytic (replication-competent, RC) adenovirus (Ad) “vectors” [1]. The first application of Ads in gene therapy was their use as replication-defective (RD) carriers to introduce foreign genes into target cells. Oncolytic Ads, on the other hand, replicate in the target tumor cells and kill these cells by virtue of replicating in them. The replication of the vector results in the release of progeny virions that can infect adjacent tumor cells and amplify the effect. In theory, the infecting oncolytic virus can spread through a solid tumor and eliminate it in the process.

The reasons for the popularity of Ad as a gene therapy vector are manifold. The biology of natural Ad infection in humans is quite well understood (reviewed in [2]), and information is available as well about experimental infection of humans with Ads and Ad vectors (reviewed in [3]). Also, the molecular biology of the virus is well studied; Ads were used as a model organism to study DNA replication, mRNA processing, oncogenic transformation, and apoptosis (reviewed in [4,5]). The replication of the virus and its effect on the host cell and the host immune system have been examined in great detail.

It is known from decades of basic research with Ads that the virus is genetically stable, and methods for engineering Ad vectors and producing of large quantities of high-titer stocks have been established. The relatively small double-stranded DNA genome lends itself for easy manipulation, either directly or cloned into bacterial plasmids [6,7]. Ads infect a wide variety of cells, quiescent or replicating, and the integration of viral DNA into the host genome is rare. Further, human type 5 Ad (Ad5), the serotype most frequently used as the parental virus for vector construction, causes asymptomatic or mild, self resolving illness in infants that results in long-term immunity to the virus [2]. Ad5 causes little, if any disease in immunocompetent adults. Thus, the danger to the treated patient is relatively low, as is the chance of an epidemic started from vector shed from the patient.

To increase the safety of Ad vectors, various modifications have been introduced to restrict vector replication to tumor cells. Most of these modifications exploit defects in certain signal transduction pathways in cancerous cells, e.g. delete viral genes regulating pro-apoptotic cellular functions known to be inactive in tumor cells, replace promoters for essential viral genes with promoters that are active only in tumor cells, or disable the vector’s capacity to promote the infected cell to a proliferative state (reviewed in [8]). Some oncolytic Ads embody a combination of these methods to achieve more stringent control of replication.

## 2. Preclinical studies with oncolytic Ads

In the past one and a half decades, many laboratories have worked to construct oncolytic Ad vectors and evaluate them in preclinical studies and clinical trials. Preclinical results with the efficacy of oncolytic Ads are promising: the vectors impeded the growth of the tumors or even completely eliminated several treated tumors in animal models [9–13]. Tumors of various origin were tested in subcutaneous, disseminated, and orthotopic models, with the vector injected intratumorally, intracavitally, or intravascularly. Further, oncolytic Ads were reported to be remarkably safe in these animal studies. To elicit toxicity, researchers injected the virus intravenously in large doses [11,14]. The results of these experiments have been extensively reviewed [15–18]. As an example, we discuss results with ONYX-015 (*dl*1520) [9] because this is the oncolytic Ad with the most extensive literature. Further, ONYX-015 was the first replication competent anti-tumor Ad vector tested, and as such, still serves as a benchmark for efficacy and safety for other oncolytic Ads with enhanced anti-tumor efficacy.

The original underlying rationale for the design of ONYX-015 is that since nearly all tumors have defective p53 pathways, E1B 55K, the anti-apoptotic viral protein responsible for binding and inactivating p53 and thus preventing virus-infected cells from undergoing apoptosis, is dispensable in cancer cells. Thus, with ONYX-015, the gene for the E1B 55K protein is deleted. Therefore, ONYX-015 was expected to be able to replicate in p53^−^ tumor cells, but induce apoptotic cell death and abortive infection in p53^+^ normal cells. Although ONYX-015 was shown to replicate preferentially in cancer cells, later publications disproved this proposed mechanism for cancer specificity [19]. It was demonstrated that rather than distinguishing cancer cells from normal cells by their p53 status, the cancer specificity of ONYX-015 stems from a defect in the export of Ad mRNA from the nucleus (which is another function of E1B 55K) [20], which is complemented by the upregulation of heat-shock protein expression in many tumor cells [21]. Upon intratumoral, intravenous, or intraperitoneal injection, ONYX-015 replicated in and suppressed the growth of subcutaneous human tumor xenografts in nude mice [9–11]. The anti-tumor efficacy of the vector increased when virotherapy was combined with the administration of chemotherapeutic agents, resulting in complete regression of tumors [22]. When injected intratumorally, ONYX-015 titers increased 100- to 1000-fold over 72 h, and vector spread within the tumor was strongly influenced by the original distribution and by the tumor microenvironment [23]. In an experiment in which cancer cells mixed with cancer cells infected with the vector at various ratios were used to generate subcutaneous tumors, preinfection of 5% of cells was sufficient to prevent tumor formation [23]. With mice, no toxicity was reported with intratumoral injection of ONYX-015. Intravenous injection of the vector caused liver toxicity with a maximum tolerated dose of 1.7 × 10^9^ plaque forming units, fractionated into five daily doses [11].

Much of the results obtained with ONYX-015 can be generalized for the oncolytic Ad field. Results with the newer vectors with enhanced anti-cancer properties will be discussed in the appropriate sections later in the article. However, certain concerns with the animal models used to obtain the data will be reviewed below.

In preclinical efficacy studies, most data have been gathered using the human xenografti-mmunodeficient (nude, SCID, *etc*.) mouse model. With this model, human tumor cells, most often from permanent cell lines, are injected to generate tumors in the mice, which allow the growth of these tumors because of the mouse’s immunodeficient status. The oncolytic Ad can be injected intratumorally or systemically, and the efficacy of the vector is determined by measuring the progression of the tumor. Tumor growth and/or vector distribution can be monitored by *in vivo* imaging techniques. The need for the highly artificial nude mouse model stems from Ads species-specific nature, *i.e*., that oncolytic Ads of human origin replicate poorly in standard laboratory rodents. With this system, the virus can replicate in the human tumor xenograft, but not in the normal organs of the host. Also, as the mice used are immunodeficient, the immune response to the vector and the tumor cannot be investigated. For safety studies, the test animals of choice have been C57BL/6 mice, probably because this mouse strain was extensively used for testing the safety of RD gene therapy vectors. Using mice, one can study the immediate toxicity caused by host response to the input virus capsids (reviewed in [24]). However, mice have a limited utility to test virus replication in normal organs and toxicity resulting from virus replication.

To overcome the shortcomings of using mice as an animal model, we [12,25] and others [26,27] have adopted a permissive, immunocompetent animal model based on the Syrian hamster. Human Ad5 replicates in several hamster cell lines, reaching a burst size between one-tenth and one-hundredth of the burst size in the human A549 lung cancer cell line, which is one of the most permissive cell lines available [25]. *In vivo*, Ad5 and Ad5-derived vectors replicate in various organs of the hamster, most prominently in the liver. The hamster cell lines can be used to form tumors in immunocompetent animals. In this model, we demonstrated that immunocompetent animals quickly develop immunity against the vector, and that this immunity has a deleterious effect on the efficacy of an oncolytic Ad [28].

In addition to anti-tumor activity, hamsters offer a good model to test the biodistribution [29] and toxicity [14] of the RC Ad vectors. After intravenous injection into hamsters, Ad5-based vectors distributed widely to all tested organs. Up to a week after injection, the DNA of a RC Ad vector was more abundant than that of a RD vector, and live, infectious RC but not RD vector could be isolated from the lung and liver of infected hamsters. The viral DNA content of the tested organs declined steadily afterward, but it could be detected in several organs even one year after virus injection. Surprisingly, at this late time, the same amount of RD and RC Ad DNA could be recovered from the animals. As no live RD vector was recovered from the animals 24 h after injection, the most likely explanation is that the real-time PCR assay detected naked viral genomes, or genome fragments, which persist in the tissues. These data point out the limitations of real-time PCR; for RC vectors, assaying for infectious vector results in more meaningful data. Supporting the findings of the biodistribution experiment, in hamsters, intravenous injection of the RC vector caused more liver damage than that of the RD vector [14]. No such difference was observed in mice, which indicates that the toxic effects of RC Ads are at least partially due to virus replication.

Another interesting result gained from the hamster studies is that immunocompetent animals cleared infectious Ads by approximately one week post injection and completely recovered from virus toxicity by four weeks post injection [14,29,30]. In contrast, large amounts of live virus could be recovered from the liver of immunosuppressed hamsters seven days post injection, and about 50% of these animals succumbed to the infection [30], indicating that the host immune system plays a major role in eliminating Ad infection. This finding has major implications for the efficacy and safety of RC Ad vectors, as it will be discussed below.

## 3. Clinical studies with oncolytic Ads

Most clinical trials conducted with oncolytic Ad vectors have been Phase I dose escalation safety studies [31], but a few vectors entered more advanced stage Phase II and Phase III trials. The bulk of the data reported deals with the safety of the vectors. Thus far, no dose limiting toxicity has been reported with any of the trials, and adverse events were mild, allaying safety concerns about oncolytic Ads. Generally, it is believed that when the vectors were used as a single modality, efficacy was modest, but much better results were achieved when treatment with the vectors was combined with radiation treatment or chemotherapy. However, no solid conclusions can be drawn about the efficacy of these vectors as such data are much more scarce and harder to interpret because most of these trials were designed as dose escalation safety studies, and assessing vector efficacy was only a secondary goal. Besides, some clinical symptoms (the swelling of the tumors after vector injection, see below) may have been misinterpreted, further skewing the results.

The vector with the most published clinical data is ONYX-015, which was evaluated in Phase I and II trials for a variety of indications, using intratumoral, intravenous, intra-arterial, and intraperitoneal routes (reviewed in [32,33]). An oncolytic Ad virtually identical to ONYX-015, named H101 (Oncorine), was approved in China in 2005 for treatment of head and neck squamous cell carcinoma [34–37]. ONYX-015 was reported to be very safe; no dose-limiting toxicities were reached even in a study in which 3 × 10^11^ plaque forming units (PFU) of ONYX-015 was injected intravenously, followed by five intratumoral injections [38]. The most-often reported adverse effect was a flu-like symptom, observed in most patients. In these trials, the efficacy of the vector was moderate [32,33]. The most promising data with ONYX-015 regarding its anti-tumor efficacy were reported by Dr. Tony Reid and colleagues. Dr. Reid treated colorectal metastases to the liver with injection of ONYX-015 directly into the hepatic artery in combination with 5’-FU/leucovorin, resulting in tumor regression in 46% of the patients [39–43]. Median survival rose to 19 months, from the expected 4–6 months survival observed in non-treated patients. Most interestingly, in some responders, tumor regression followed an acute enlargement of tumors [40–42]. While these tumors initially increased in size as detected by CT scans, PET imaging revealed that they had very low metabolic activity. This suggests that most of the tumor mass detected by CT scan might have been necrotic. With the majority of clinical trial protocols, in which PET imaging is not applied, this initial swelling would have been reported as progressive disease, missing the possible positive effect. Thus, multi-modal monitoring of the patients treated with oncolytic Ads is a must.

Similar safety and efficacy data were published with two other groups of oncolytic Ad vectors. The first group of vectors has the same *e1b-55K* deletion as ONYX-015 but also express a fusion protein consisting of a cytosine deaminase (CD) and herpes simplex virus thymidine kinase (TK) prodrug-converting enzymes (reviewed in [44]). These vectors were used to treat prostate tumors by direct injection of the vector followed by prodrug administration. In some cases, the oncolytic Ad treatment was combined with radiotherapy. The results of two Phase I clinical trials have been published with these vectors. [45–47]. The data indicate that following intra-prostatic injection the vectors were remarkably non-toxic, and have provided measurable clinical benefit to a stratum of patients (those with intermediate disease risk).

The other family of oncolytic Ad vectors with data in the public domain about clinical trials is also designed to treat prostate cancer. The replication of these RC Ads is restricted to PSA-positive (*i.e.,* prostate) cells by replacing the original viral promoters of essential viral genes for PSA and rat probasin promoters. The vectors produced low toxicity, no DLTs, no MTD at vector doses up to 10^13^ virus particles (vp) after intra-prostatic injection [48,49] and 3 × 10^12^ vp after intravenous injection [50], and some patients showed a decline in PSA levels [50].

Recently, information about the results of clinical trials with a family of oncolytic Ads has become available. In one version of the vector, named Ad5/3-Cox2L-D24, the Ad5 fiber gene was substituted with that of Ad3 to achieve CAR-independent attachment. For tumor specificity, the vector has a 24 bp deletion in the *e1a* gene, abrogating its binding to the Rb family proteins, and the expression of *e1a* is regulated by the cyclooxygenase 2 promoter [51–53]. Another version of this vector, named ICOVIR-7, has the same *e1a* deletion, wild-type *e1a* promoter, and it has an RGD peptide inserted into the HI-loop of the Ad5 fiber protein instead of Ad3 fibers [54]. The third vector in this family has wild-type *e1a* promoter, the same *e1a* deletion, wild-type fiber, and it expresses GM-CSF [55]. All the three vectors were tested in patients with various solid tumors, demonstrating low toxicity and objective responses in approximately 60% of patients.

## 4. Enhancing the efficacy of oncolytic vectors

From the data delineated above, it is clear that there is an inconsistency between the efficacy data obtained from tissue culture, animal experiments, and clinical trials. Tissue culture results do not translate directly to animals. Even when a particular vector kills a given cell line very efficiently *in vitro,* tumors formed using this cell line may not get eliminated completely. Multiple factors can cause this discrepancy between *in vitro* and *in vivo* data. Some observations indicate that virus spread within tumors is limited. After injection, oncolytic Ad-infected cells make up just a minor fraction of the tumor mass, usually clustered around the needle track, and infectious virus titers in tumors decrease rapidly [56]. Tumor architecture can be a major barrier to vector spread. In tissue culture monolayer, which is practically two-dimensional, the virus has ready access to all infectable cells; in contrast, tumors can be heavily compartmentalized. Thus, even if the vector efficiently eliminates all tumor cells within a compartment, fibrotic septa and necrotic areas may prevent its spread to the neighboring compartment of live tumor cells. In addition, in immunocompetent animals, and expectedly in patients, the immune system eliminates most of the virus in 14 days after virus injection [28].

The immune response to the vector may contribute to the worse than anticipated efficacy of oncolytic Ads in clinical trials. As discussed above, most vector efficacy data were gathered in the immunodeficient mouse-human tumor xenograft model, in which the efficacy of the vector is not affected by anti-viral immunity. Further, oncolytic Ad clinical trials usually have been carried out with terminal patients who went through multiple rounds cancer therapy. These tumors are usually very large, heavily compartmentalized, necrotic, and are refractory to conventional treatment. This is in great contrast to the small subcutaneous tumors injected in most animal experiments.

Thus, while oncolytic Ads have been proven safe without a doubt, their efficacy warrants improvement. Below we summarize the approaches researchers have taken to increase the anti-tumor effects of oncolytic Ads.

## 5. Selecting for the Ad with the best anti-tumor features

Gain-of-function mutations in the viral genome result in more efficient anti-tumor agents. One approach to generate such a vector is to start with a pool of Ads of various serotypes, which are then repeatedly passaged on cancer cell lines at low multiplicity. This procedure will select for the fastest-spreading virus. Such a procedure resulted in the isolation of an Ad3/Ad11 chimera with superior oncolytic features [57]. Alternatively, oncolytic Ad vectors with superior growth characteristics on cancer cell lines can be obtained by random mutagenesis and propagation of the resulting mutants on cancer cells, selecting for the fastest-growing isolate [58–60].

## 6. Avoiding over-attenuation of the vector

In the initial phase of vector development, the priority for designing RC Ad vectors was safety. Vector replication was restricted to cancerous cells by modifying the Ad genome. However, these modifications caused some oncolytic vectors to replicate less well in cancer cells than did the parental wild-type Ad5. Inasmuch as the premise of oncolytic virotherapy is that the vector replicates and spreads in the infected tumor, this attenuation inevitably decreased anti-tumor efficacy.

We reason that extensive attenuation of the vector (and the consequent decrease in efficacy) is not necessary. First, it has been demonstrated that Ads replicate preferentially in cells with de-regulated cell cycle [61]. Second, most clinical protocols call for intratumoral injection of the vector. The circulating neutralizing antibodies, present in most adults, are very efficient in neutralizing virions escaping into the blood stream [30,62]. Third, most oncolytic Ads are derived from species C Ads, and infections with these Ads are mostly asymptomatic in immunocompetent adults [2]. Fourth, even in the unlikely case of uncontrolled disseminated infection, anti-viral drugs can be used to inhibit Ad replication and thus limit the severity of disease [30]. To this effect, we have constructed VRX-007 (also known as INGN 007), an oncolytic Ad5-based vector without engineered attenuating features [63]. VRX-007 has the genes for the immunomodulatory E3 proteins [64] deleted, which further increases its safety. VRX-007 overexpresses the adenovirus death protein (ADP, see below) to speedup its cell-to-cell spread. We compared the efficacy of VRX-007 to another oncolytic Ad named KD3 [13]. KD3 is the parental vector of VRX-007, and it is identical to VRX-007 except that KD3 was engineered to replicate preferentially in cells with a deregulated cell cycle due to two small deletions in the *e1a* gene [65,66]. *In vitro*, VRX-007 has a broader host range than KD3 [13]. *In vivo*, the two oncolytic Ads were equally efficacious in some tumor models; however, in a challenging intravenous administration model VRX-007 clearly outperformed KD3, and was also significantly more efficacious that ONYX-015 [13]. Toxicology and biodistribution experiments validated our original tenet, inasmuch as the safety profile of VRX-007 was similar to that of Ad5 in both permissive and non-permissive animal models [14,29]. VRX-007 is presently being tested in a Phase I clinical trial for intratumoral injection into solid tumors of any indication.

## 7. Better delivery of the infecting bolus of vector

Evidently, the more that the injected vector actually reaches tumor cells, the better the expected anti-tumor efficacy. The physical delivery of RC Ads to tumor cells is made difficult by multiple factors. As discussed above, most solid tumors have a very complex architecture, with barriers preventing the spread of the vector throughout the tumor. Researchers and clinicians have been trying to circumvent this obstacle by better distributing the Ad vector within the tumor using multiple injections [67] or injecting the vector into the artery supplying the tumor [68]. Another approach is to try to modify the tumor matrix by expressing transgenes so that the matrix is more permeable for virus spread; this method will be discussed below. Treatment of superficial bladder cancer with oncolytic Ads has been contemplated [69]. It was shown that a glycosaminoglycan layer covering the inner surface of the bladder that inhibits Ad attachment. Eliminating this layer using detergents improved the infection rate and efficacy of Ad vectors [70,71].

Ad vectors can be delivered to tumors using mesenchymal stem cells (MSC) as delivery vehicles. MSCs can be productively infected with Ads [72], and were shown to home to tumors upon systemic delivery (for a review, see [73]). Thus, intravenously injected MSCs infected with oncolytic Ads seek out tumors, where they will lyse and release the vector. Ad5/3 fiber-chimeric Ad-infected MSCs homed to intraperitoneal ovarian tumors in mice after intraperitoneal injection and provided survival benefit over injection of Ad5/3 alone [72]. It was also demonstrated that when injected intravenously into orthotopic lung and breast cancer models in nude mice, MSCs infected with an Ad vector showed increased efficacy compared to the same vector injected alone, even though accumulation of the injected MSCs at tumor sites could not be demonstrated [74].

Beyond the mere physical delivery of a virus particle to a tumor cell, infection also depends on the presence of cell surface receptors. The primary receptor for Species C Ads is the Coxsackie-Adenovirus common Receptor (CAR), which, although expressed on many cell types, is low or is absent from certain tumors (e.g. bladder and ovarian cancer) [75,76], rendering these tumors poorly infectable by species C Ads. However, Ads can infect tumors in a CAR-independent manner [77,78]. Unfortunately, some of the same non-CAR-mediated processes are responsible for the predisposition of Ads to infect hepatocytes [79]. Although it has been shown that intravenous administration of an oncolytic Ad can be efficacious [52], the liver tropism of oncolytic Ads is widely believed to be a hindrance for systemic delivery of the vector; besides the liver toxicity, the anti-viral efficacy decreases because only a fraction of the vector reaches the intended target. Researchers tackle these obstacles by either genetically- or chemically-modifying the capsid of the vector, and thereby targeting the vector to tumors through selective transduction. Transductional targeting of oncolytic Ads by genetic modification of the fiber knob, using bi-specific adapter molecules, and chemical modification of the capsid is the subject of many excellent reviews [80–83]. Here we will review some of the methods researchers used to target vector binding to specific cells.

To extend the host range of an Ad vector to cells with little or no CAR expression, researchers swapped the fiber of a Species C-based vector to that of a Species B Ad [84]. Species B Ads use cell surface proteins other than CAR, such as CD46 [85] or CD80/86 [86] to infect the host cell. These receptors are present on some cell types on which CAR is absent (most notably ovarian cancer cells), thus allowing these Species C/B pseudotyped vectors to infect cells that are poorly infectable with Ad5-based vectors. Another approach changes only the receptor-binding surfaces of the fiber by inserting short peptide sequences, like a polylysine tract or the arginine-glycine-aspartate (RGD) motif of penton base, into the HI loop [87] or carboxyl terminus of the molecule [88]. Vectors carrying such modifications were able to infect cells with heparan sulfate glucosaminoglycans [88] and a_v_β_3–5_ integrins [87] on their surface. While several laboratories generated Ad vectors with more specific peptide ligands in their capsid proteins [89–91], it was found to be very difficult to predict if incorporation of a given ligand will target the vector to the desired cells [92].

Others constructed virions bearing chimeric fibers that contain domains of structurally homologous proteins from other, phylogenetically distant viruses like bacteriophage T4 or reovirus [93,94]. To provide targeting specificity, they attached single-chain antibodies [95] or CD40 ligand [96] to these synthetic fibers.

Genetic capsid modifications like the ones mentioned above have resulted in extending the host range of the parental vector. However, they did not change the vector’s predilection to infect the liver when administered systemically. *In vivo*, Ads can infect cells in a CAR-independent way through some plasma proteins binding to both the viral capsid and certain cells, serving as adaptor molecules to facilitate the infection of these cells. Most importantly, coagulation factor X (FX) binds both the Ad hexon protein and heparan-sulfate proteoglycans on hepatocytes [97,98]. As this binding proves to be a major factor behind Ads’ tropism for the liver, the modification of fiber only is insufficient to prevent the vectors’ natural tropism. Mutating the recently identified factor X binding sites on the Ad5 hexon proteins seems to have solved this problem [99].

Chemical modification of the virions can result in altered tropism as well. The fundamental difference between this method and the genetic modifications discussed above is that while the genetic modifications are present in the progeny virions as well, chemical modification affects only the input bolus of virions. The same caveat applies to all similar designs such as Ad-liposome complexes [100,101], and bispecific ligands [102,103]. With chemical modification, the virion is coated with a hydrophilic polymer, most often with polyethylene glycol (PEG) or poly-N-(2-hydroxypropyl) methacrylamide (pHPMA) (reviewed by Kreppel [104]). This “shield” will protect the vector from circulating antibodies, allowing for repeated administration of vector. Shielding with PEG inhibits CAR-mediated binding to cells *in vitro* [105]. However, it generally does not affect CAR-independent infection of liver *in vivo*, with the exception that high molecular weight PEG coated virions could not exit through the fenestrae of the liver endothelium and were physically unable to gain access to hepatocytes [106,107]. Ligands for receptors present on tumor cells or tumor endothelium (e.g. EGF, FGF-2, VEGF) were coupled to PEG-coated virions to specifically target the vector to tumors [108–110].

## 8. Preventing the anti-viral immune response

The recent use of immunocompetent animal models by a number of investigators has allowed the effect of the host immune system on the oncolytic Ad vector-infected tumor to be studied in detail. The immune system could have at least two effects on the efficacy of oncolytic Ad vectors: the developing anti-virus immunity can eliminate the vector, thus decreasing efficacy, or the infection of the tumor by the vector can change the otherwise immunosuppressive environment of the tumor and induce the development of anti-tumor immune response, thus enhancing efficacy. We have addressed these questions by employing the Syrian hamster animal model.

We compared the efficacy of VRX-007 injected intratumorally into syngeneic kidney cancer tumors in immunocompetent and immunosuppressed hamsters. We demonstrated that the immunocompetent hamsters quickly developed anti-vector immunity that significantly decreased the efficacy of VRX-007 [28]. In immunocompetent hamsters, the vector was effective initially in suppressing the growth of tumors following intratumoral injection, but then as anti-vector immunity developed, the efficacy was decreased and the tumors resumed growth typically after 2–4 weeks following vector injection. Repeated injection of the vector after tumors had started growing again did not decrease the growth rate. In contrast, in immunosuppressed animals the efficacy of the vector was sustained [28,111,112]. To explain the apparent contradiction with results obtained in clinical trials, *i.e.* that circulating neutralizing antibodies to Ad have no effect on the efficacy of intratumorally injected vector [50,54], we tested the efficacy of VRX-007 in kidney tumor-bearing hamsters that were either naïve regarding exposure to Ad or were immunized with the virus, and then either left untreated or immunosuppressed [111]. Hamsters that were immunized with Ad had very high anti-Ad neutralizing antibody levels to start with, but even naïve, immunocompetent animals produced neutralizing antibodies by seven days after intratumoral injection of the vector, and these antibodies were present in the tumor as well [111]. Consistently, there was no significant difference between the efficacy of VRX-007 in these two groups of animals, *i.e.*, the vector suppressed the growth of tumors following intratumoral injection for 2–3 weeks, but then the tumor growth resumed. However, when the vector was injected into immunosuppressed animals, the efficacy of VRX-007 was much decreased in the hamsters that were immunized before immunosuppression and had neutralizing antibodies at the time of VRX-007 injection compared to the naïve, immunosuppressed hamsters that did not have antibodies at the start of treatment and had no potential to raise them. Thus, we conclude that naïve animals, and most likely patients, develop anti-Ad immunity very quickly, which will impede the anti-tumor efficacy of an oncolytic Ad vector, and that the presence of pre-existing immunity does not have a great impact on anti-tumor efficacy [111]. Our data also indicate that repeated cycles of vector injection into immunocompetent patients will probably not increase the efficacy of the treatment. However, this interpretation does not, and cannot account for the great variability within patient populations, which can introduce unknown factors that might influence the immune response and vector efficacy.

Pre-existing immunity, however, can have a beneficial effect on the safety of oncolytic Ads. Previous exposure to Ad or passive immunization using hyperimmune serum can prevent the dissemination of the vector to normal organs, and it can prevent lethal intravenous challenge with Ad5 even in immunosuppressed animals [62].

Besides of scientific interest, these finding may have some relevance in designing cancer treatment regimes as well. As many chemotherapeutic drugs are immunosuppressive, it could be feasible to devise a protocol to take advantage of this effect, *i.e.,* start the chemotherapy treatment before injecting the Ad vector. In fact, it is conceivable that the enhanced efficacy of oncolytic Ads in combination with chemotherapy resulted from immunosuppression of the patient.

## 9. Armed oncolytic Ad vectors

While the premise of cancer therapy with oncolytic Ads is that the vector will replicate in tumor cells and kill them through the lytic replication cycle, this effect can be enhanced by expressing anti-cancer therapeutic molecules (*i.e.* “arming” the vector). This can partially overcome the attenuation of vectors that have lost their potency because of multiple genetic modifications implemented to achieve tumor-specific replication. The most common site to insert transgenes is in place of the deleted E3 region, where one can take advantage of the native viral promoters and differential splicing in the viral genome to express these genes either in the early or the late phase of Ad replication [113]. Alternatively, transgenes can be expressed from strong constitutive or inducible foreign promoters. As the infected cell is expected to be killed by the oncolytic Ad, therapeutic molecules in armed oncolytic Ads are usually secreted or have a bystander effect to affect cells beyond the one that is infected by the virus. There are numerous therapeutic molecules expressed from oncolytic Ads; many of them were adopted from cancer therapy treatment with RD viral vectors. These molecules can be grouped by function; the groups are discussed below.

## 10. Molecules enhancing vector spread

As the proposed mechanism of action for oncolytic Ads relies on the production of progeny virus and the spread of infection through a solid tumor, it is clear that any increase in vector spread will result in an increase in anti-tumor efficacy. To speed up the first step in vector spread, namely the egress of progeny virions from the infected cells, researchers have over-expressed the Ad ADP protein [13,63,65,114]. Another way to facilitate virus release from infected cells is to introduce a deletion into the *e3 gp19K* gene [58]. Others employed a fusogenic protein from an enveloped virus which causes the membrane of the infected cell to fuse with neighboring cells, resulting in formation of large syncytia, thus enhancing cell-to-cell spread [115]. Once the infected cells have lysed, virus is released to the intercellular matrix and can infect nearby cells. As discussed above, tumor architecture that contains necrotic areas and fibrotic stroma often hampers the spread of the vector beyond the immediate vicinity of infection site [56]. To make the tumor microenvironment more permeable, a research group expressed the peptide hormone relaxin from their oncolytic Ad vector [116,117]. Relaxin increases the production of certain matrix metalloproteases and decreases the production of collagen. The intratumoral spread and the anti-tumor efficacy of the relaxin-expressing vector were significantly better than those of the parental vector.

## 11. Prodrug-converting enzymes

Prodrug-converting enzymes, enzymes that convert non-toxic prodrugs to the active form (e.g. thymidine kinase, cytosine deaminase, nitroreductase [118,119]), have been used in RD vectors for a long time, and can be adapted readily to use in oncolytic vectors. With this kind of therapy normal cells do not get exposed to the toxic product, because although the prodrug is administered systemically, it is processed into the toxic drug only in the vector-infected tumor cells. This ensures high local concentration of the drug, because the converted drug then can leave the infected cell and be taken up by neighboring cells, thereby causing a local bystander effect. Besides being toxic to cancer cells, thymidine kinase can be also used to image the vector-infected tissues by injecting a PET or SPECT tracer substrate [120]. The timing of the administration of the prodrug is critical, because many of their products are chain-terminator nucleotide analogues, and will prevent virus replication. Thus, these can be considered “suicide gene” safety measures, which, when the prodrug is injected, will stop runaway virus infection.

## 12. Immunomodulatory molecules

Tumor cells have evolved multiple tactics to evade the immune system: they can express immunosuppressive cytokines, down-regulate Major Histocompatibility Class I molecules, and even express Fas ligand to kill reactive cytotoxic lymphocytes. Thus, the tumor microenvironment prevents the immune system from eradicating tumors, even those that express tumor antigens [121]. Infection of the tumor with Ads elicits a strong inflammatory immune response [122], which may overcome this immunosupression. This immune response can be improved further by expressing cytokines and chemokines that are being investigated in cancer immunotherapy (reviewed in [123]) from the virus. The immunomodulatory transgenes tested so far include GM-CSF to stimulate the production of antigen presenting cells [124,125], Fas ligand and IL-27 to facilitate antigen presentation on dendritic cells (DCs) [126], interferons α, β, and γ for direct anti-tumor activity and to induce a proinflammatory effect [127–129], the chemokine RANTES for recruiting DCs and T lymphocytes [130], and IL-12 to activate T lymphocytes [131]. To overcome the defects in antigen presentation that many tumor cells have acquired, researchers developed an oncolytic Ad that expresses the chaperone hsp70 in the tumor cell [132]. The hsp70 molecule will be “loaded” with the peptides of the infected cell, among them peptides that could serve as epitopes of tumor antigens. When the infected cell is lysed by the RC Ad vector, the DCs will take up the peptide-chaperone complexes and present the peptides on Class II molecules, thus raising an immune reaction to the tumor.

Most of the experiments with immunomodulatory molecules expressed from oncolytic Ads were performed *in vitro*, or using human xenografts in immunodefective mice, in which the transgene may not be functional, or using immunocompetent mice with syngeneic tumors, in which the vector does not replicate. With the recent development of immunocompetent, permissive animal models, researchers can test these vectors in a more adequate system. An oncolytic Ad expressing human interferon α showed increased efficacy against syngeneic kidney tumors in Syrian hamsters [133]. As an added benefit, the expression of interferon α dramatically decreased vector toxicity [129,133]. Another research group demonstrated that a human GM-CSF expressing oncolytic Ad eradicated subcutaneous pancreatic tumors in hamsters, and that the hamsters that became tumor free after treatment with the vector developed anti-tumor immunity [55].

These transgenes will presumably increase the host immune response to the infecting vector as well; although this will hasten the elimination of the vector and thus work against the original premise of how oncolytic viruses work, still, the end result may be enhanced oncolysis.

## 13. Molecules with direct anti-tumor effect

Cytotoxic molecules can be expressed in the oncolytic Ad-infected tumor in order to ensure high intratumoral concentration and at the same time avoid exposing the normal cells of the body to these highly toxic substances.

Several oncolytic Ad vectors expressing apoptosis-inducing death ligands like TNFα, Fas ligand, and TRAIL have been described [126,133–135]. This approach, similarly to RC Ads combined with immunotherapy, may have a deleterious effect on vector replication and spread. The secreted protein can kill uninfected tumor cells surrounding the infected cell, and thus creating a “firewall” of dead cells that can prevent virus spread.

## 14. Anti-angiogenic agents

Tumor neovasculature presents a very promising target within the tumor. The uncontrolled growth of tumors causes hypoxic conditions, which in turn stimulates the formation of blood vessels, further aided by the secretion of pro-angiogenic factors by some tumors. Interfering with tumor angiogenesis will deprive the tumor of nutrients and oxygen (reviewed in [136]). As neo-endothelium expresses high levels of αvβ3 integrins [137], one of the cellular co-receptors for Ad binding, Ads are naturally targeted to newly formed blood vessels. This effect can be further enhanced by expressing anti-angiogenic molecules including endostatin [138], antagonists of VEGF [139,140], certain cytokines, (e.g. type I interferons [127,129], and MDA-7/IL-24 [141]) from oncolytic Ads. There is a possibility of synergism with this latter approach, as the target of the transgene is different from the cancer cells themselves that are being destroyed by virus replication.

## 15. Conclusion

It has been proven through numerous preclinical and clinical experiments that oncolytic Ad vectors are safe to use, especially with patients with pre-existing immunity. The vectors’ efficacy can be increased through multiple methods that include modifying the vector itself, employing better delivery techniques, or preventing the elimination of the vector by the host immune system. As the mechanism of action of oncolytic Ads is different from that of conventional cancer treatment, RC Ad treatment can be incorporated into existing treatment regimes, which will likely increase efficacy of treatment over a single modality. Thus, oncolytic Ads will be an additional treatment option to combat cancer.
